# Successful treatment with endoscopic transpapillary drainage for gallbladder perforation associated with steroid treatment for interstitial pneumonia (with video)

**DOI:** 10.1002/jgh3.12397

**Published:** 2020-07-30

**Authors:** Yujiro Kawakami, Kazuya Suzuki, Masakazu Akahonai, Takakazu Miyake, Masahiro Taniguchi, Hiroshi Nakase

**Affiliations:** ^1^ Department of Gastroenterology and Hepatology Sapporo Medical University School of Medicine Sapporo Japan; ^2^ Department of Gastroenterology Kushiro City General Hospital Kushiro Japan

**Keywords:** a high‐risk surgical patient, acute cholecystitis, endoscopic gallbladder stenting, endoscopic transpapillary drainage, gallbladder perforation

## Abstract

This case report highlights the clinical efficacy of endoscopic transpapillary drainage for gallbladder perforation in a high‐risk surgical patient with a history of steroid treatment for interstitial pneumonia. The usefulness of endoscopic transpapillary gallbladder drainage in high‐risk surgical patients with acute cholecystitis has not been established. In difficult cases of emergent surgery, such as described here, endoscopic transpapillary drainage is a promising method to manage gallbladder perforation and acute cholecystitis recurrence.

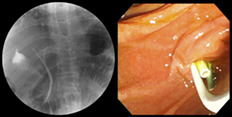

A 74‐year‐old woman who had been treated with prednisolone for interstitial pneumonia was referred to our department because of complaints of severe abdominal pain and high fever. Physical examination revealed tenderness in the right upper quadrant with positive Murphy's sign. Blood tests showed elevated white blood cell count and C‐reactive protein level. Computed tomography (CT) revealed an enlarged gallbladder with pericholecystic inflammation (Fig. 1a); therefore, she was diagnosed with acute cholecystitis (AC). Because she was a high‐risk surgical patient, we started antibiotics treatment. On day 2 of hospitalization, physical examination revealed peritoneal irritation without Murphy's sign. CT showed the appearance of ascites with the reduction of gallbladder swelling (Fig. 1b). Physical examination and imaging findings suggested gallbladder perforation. We emergently performed endoscopic retrograde cholangiopancreatography (ERCP) (Video Clip S1, Supporting information), demonstrating leakage of the contrast medium from the wall of the gallbladder fundus (Fig. 1c). Endoscopic biliary stenting and endoscopic nasobiliary drainage (ENBD) were performed to decompress the pressure of the biliary tract together with endoscopic pancreatic stenting to prevent post‐ERCP pancreatitis (Fig. 1d). After these procedures, her symptoms dramatically improved. On day 12 of hospitalization, we confirmed no leakage from the gallbladder into the abdominal cavity (Fig. 1e). We removed the ENBD tube and performed endoscopic gallbladder stenting to prevent AC recurrence (Fig. 1f). She had no AC recurrence under treatment with oral prednisolone.

**Figure 1:**
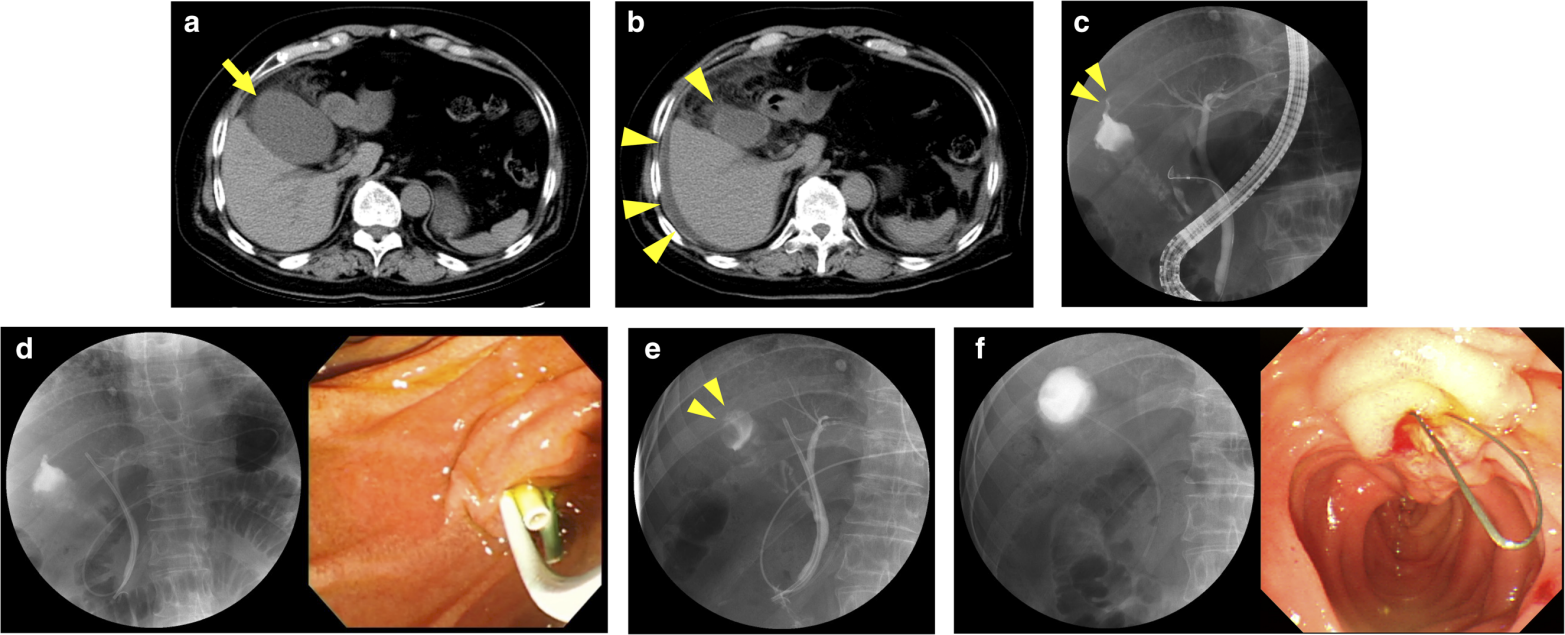
(a) Computed tomography (CT) revealed the enlarged gallbladder with pericholecystic inflammation (arrow). (b) CT showed the appearance of ascites with the reduction of gallbladder swelling (arrowhead). (c) Endoscopic retrograde cholangiopancreatography demonstrated leakage of the contrast medium from the wall of the gallbladder fundus (arrowhead). (d) A 7‐Fr plastic stent (PS) was placed in the right intrahepatic bile duct, and a 5‐Fr endoscopic nasobiliary drainage catheter was placed in the left intrahepatic bile duct. A 5‐Fr PS was placed in the main pancreatic duct. (e) Fluoroscopic image revealed no leakage from the gallbladder into the abdominal cavity (arrowhead). (f) A 5‐Fr single pigtail stent with thread (Biliary stent with thread; CX‐T stent; Gadelius Medical, Tokyo, Japan) was placed in the gallbladder.

The standard treatment for perforated cholecystitis is emergent cholecystectomy.^1^ However, several reports indicated that cholecystectomy was occasionally difficult in high‐risk elderly patients with any comorbidities.^2^, ^3^ The usefulness of endoscopic transpapillary gallbladder drainage in high‐risk surgical patients with AC has not been established.^4^, ^5^ In the difficult cases of emergent surgery, such as we experienced, endoscopic transpapillary drainage could be a promising method to manage gallbladder perforation and AC recurrence.

## Supporting information


**Video Clip S1** Successful treatment with endoscopic transpapillary drainage for gallbladder perforation.
